# Characterization of phytohormone and transcriptome reprogramming profiles during maize early kernel development

**DOI:** 10.1186/s12870-019-1808-9

**Published:** 2019-05-14

**Authors:** Chuanyu Ma, Bo Li, Lina Wang, Ming-liang Xu, E. Lizhu, Hongyu Jin, Zhicheng Wang, Jian-rong Ye

**Affiliations:** National Maize Improvement Center, China Agricultural University, 2 West Yuanmingyuan Road, Beijing, 100193 People’s Republic of China

**Keywords:** Phytohormone, Defense response, Maize early kernel development, Transcriptome reprogramming, Auxin

## Abstract

**Background:**

During maize early kernel development, the dramatic transcriptional reprogramming determines the rate of developmental progression, and phytohormone plays critical role in these important processes. To investigate the phytohormone levels and transcriptome reprogramming profiles during maize early kernel development, two maize inbreds with similar genetic background but different mature kernel sizes (ILa and ILb) were used.

**Results:**

The levels of indole-3-acetic acid (IAA) were increased continuously in maize kernels from 5 days after pollination (DAP) to 10 DAP. ILa had smaller mature kernels than ILb, and ILa kernels had significantly lower IAA levels and significantly higher SA levels than ILb at 10 DAP. The different phytohormone profiles correlated with different transcriptional reprogramming in the two kernels. The global transcriptomes in ILa and ILb kernels were strikingly different at 5 DAP, and their differences peaked at 8 DAP. Functional analysis showed that the biggest transcriptome difference between the two kernels is those response to biotic and abiotic stresses. Further analyses indicated that the start of dramatic transcriptional reprogramming and the onset of significantly enriched functional categories, especially the “plant hormone signal transduction” and “starch and sucrose metabolism”, was earlier in ILa than in ILb, whereas more significant enrichment of those functional categories occurred at later stage of kernel development in ILb.

**Conclusions:**

These results indicate that later onset of the significantly enriched functional categories, coincide with their stronger activities at a later developmental stage and higher IAA level, are necessary for young kernels to undergo longer mitotic activity and finally develop a larger kernel size. The different onset times and complex interactions of the important functional categories, especially phytohormone signal, and carbohydrate metabolism, form the most important molecular regulators mediating maize early kernel development.

**Electronic supplementary material:**

The online version of this article (10.1186/s12870-019-1808-9) contains supplementary material, which is available to authorized users.

## Background

Maize kernel development begins with double fertilization of a haploid egg cell and dikaryotic central cell to produce two filial structures: a diploid embryo and triploid endosperm. This complex biological process can be divided into three phases: early development, reserve filling and dehydration, and phyohormones play vital roles during this process [1–4]. The first 10 days after pollination (DAP) is the early maize kernel development stage, in which a few key developmental processes occur [3]. During 0–3 DAP, the endosperm undergoes nuclear divisions without cellularization to form a large coenocyte. Then followed by endosperm cellularization and an intense period of cell division, this early mitotic phase lasts until 8–12 DAP in the central region, resulting in rapidly growing maize endosperm that filled entire seed cavity. This mitotic cell division phase is largely responsible for generating the final population of endosperm cells [4]. At 9 DAP, the kernel undergoes the differentiation peaks of cell types in the embryo and endosperm, and storage product synthesis and accumulation initiates at ∼10 DAP in maize endosperm. At 15 DAP, the embryo-surrounding region (ESR) disappears and synthesis of endosperm starch and storage proteins peak [4].

After endosperm cellularization, the endosperm differentiates into four main cell types, starchy endosperm (SE), basal endosperm transfer layer (BETL), aleurone layer (AL) and ESR, each cell type has unique hormonal characteristics, cellular morphologies and gene expression patterns [3]. Although cytokinins (CKs) promote endosperm cell divisions, maximal CK content is reached 1–2 days before the peak in endosperm mitotic activity [5], the mutant *mn1* kernels showed slightly higher levels of CK than the *Mn1* kernels during kernel development [6]. Auxin has fundamental roles in plant vegetative and reproductive growth and development. During *Arabidopsis* seed development, auxin response is observed in the earliest stage embryo [7], while auxin is not detected in maize kernel until the transition stage (~ 7 DAP) [8]. The developing maize endosperm synthesizes large quantities of IAA, and IAA concentration in kernel abruptly increased from 9 to 11 DAP [9]. High auxin concentrations are detected in the highly specialized BETL tissue, which contains deep cell ingrowths at the basal surface that facilitate nutrient uptake from the apoplastic space in the placenta chalazal region [10]. Auxin is hypothesized as the signal that triggers the transition to the filling stage, as it reaches a maximal concentration in whole kernels at ~ 12 DAP [11]. The *mn1* kernels are most notably deficient for indole-acetic acid (IAA), where both free and IAA sugar conjugate forms were at least 10-fold reduced in the mutant throughout kernel development [11]. Seven maize *YUC* genes, which encode proteins containing the flavin-binding monooxygenase-like domain that converts indole-3-pyruvate (IPA) to IAA, are primarily expressed in developing kernels [12–14]. *De18* (*defective endosperm18*) encodes the endosperm-specific *ZmYUCCA1*. The endosperm of mutant *de18* maize kernels displays impaired IAA biosynthesis, large reductions in free IAA levels throughout endosperm development, and finally approximately ~ 40% less dry mass than wild type, suggesting a disruption of nutrient uptake [15].

Plants have evolved to efficiently integrate external and internal cues into finely tuned growth programs that provide optimum fitness under diverse environmental conditions. Phytohormones such as auxin, salicylic acid (SA), jasmonic acid and its derivatives (JAs), integrate endogenous developmental cues with environmental signals to regulate plant growth and defense. Crosstalk between growth hormones (auxin) and defense hormones (SA and JAs) mediate trade-offs between plant growth and defense networks [16]. SA is a critical signal for activating disease resistance in plant. JAs are lipid-derived stress hormones that regulate plant adaptations to biotic stresses (herbivore attack and pathogen infection) and abiotic stresses (ozone, ultraviolet radiation, high temperature, and freezing) [17–19]. Currently, there is limited information about the role of SA and JA in maize kernel development. Auxin signaling negatively affects plant innate immunity. Host susceptibility responses to pathogen infection are often mediated by induced auxin biosynthesis or modulated auxin signaling [16, 20]. Auxin antagonizes SA signaling in plant growth and defense, and SA represses auxin signaling by inhibiting auxin biosynthesis, uptake, sensitivity, and TIR1/ABF F-box receptor complex expression. Higher SA levels reduce the pool of active IAA, thereby prioritizing defense responses over growth; constitutively active SA-induced defense responses lead to retarded plant growth phenotypes [21–23]. JA synergistically interacts with auxin to benefit some plant pathogens [24, 25]. To enhance sustainable plant defense responses, efforts should be made to minimize the negative effects of auxin on immunity and reduce SA- and JA-mediated growth losses.

Early plant immunity is characterized by an oxidative burst, which induces the accumulation of reactive oxygen species (ROS) to suppress pathogen invasion or induce plant programmed cell death or hypersensitive response [26, 27]. Pathogenesis-related (PR) proteins are the hallmark of the induced defense response during plant-pathogen interactions, and their expression is associated with plant basal immunity [28]. The defense response cascade also produces along with the accumulation of antimicrobial compounds, peroxidases, cytochrome P450 proteins (CYPs), and glutathione-S-transferases (GSTs) [29]. The number and abundance of the defense-related products (PR1, PR5, PRm3, and PRm6), oxidative stress–related enzymes (peroxidase, catalase, superoxide dismutase, and ascorbate peroxidase) and the speed of defense responses determine the success of pathogen infection [30]. WRKY family members have critical roles in plant immunity, senescence, and SA- and ABA-mediated plant defense and abiotic stress tolerance [31]. OPAQUE11 (O11) is an important regulator for growth–defense tradeoffs during maize kernel development, O11 coordinates cell development, storage nutrient metabolism, and stress responses during maize endosperm development by directly regulating many stress response genes, such as the *PR* gene, *peroxidases*, *GST*, and stress response transcription factor (TF) genes such as *ZmWRKY53*, *ZmWrky40*, and *ZmJAZ1* [32].

Maize is a globally important crop, its mature kernel size is determined during early kernel development. During kernel development, patterns of kernel weight accumulation show an earlier onset, slower rate, and earlier termination of grain filling in small kernels relative to large kernels, and transcriptome patterns reveal an earlier onset of key genes in small kernels, while similar maximum transcription levels in large kernels at later stages [33]. However, a permanent and efficient defense response to environmental stresses is required for the sessile plants to sustain their growth, the simultaneous activation of defense and maintenance of growth are critical for kernel development. To investigate phytohormone (IAA, SA, and JA) mediated growth-defense responses and transcriptome reprogramming profiles during maize early kernel development, we used two maize inbreds with similar genetic background but different mature kernel sizes, and collected their developing kernels at 5 DAP (early mitotic phase), 8 DAP (before abruptly increased IAA concentrations), and 10 DAP (peak cell type differentiation in the embryo and endosperm and initiation of storage product synthesis and accumulation in the endosperm) to generation the data on phytohormone contents and transcriptome profiles.

## Results

### ILa kernels at 10 DAP had lower IAA levels and higher SA levels than ILb kernels

**Fig. 1:**
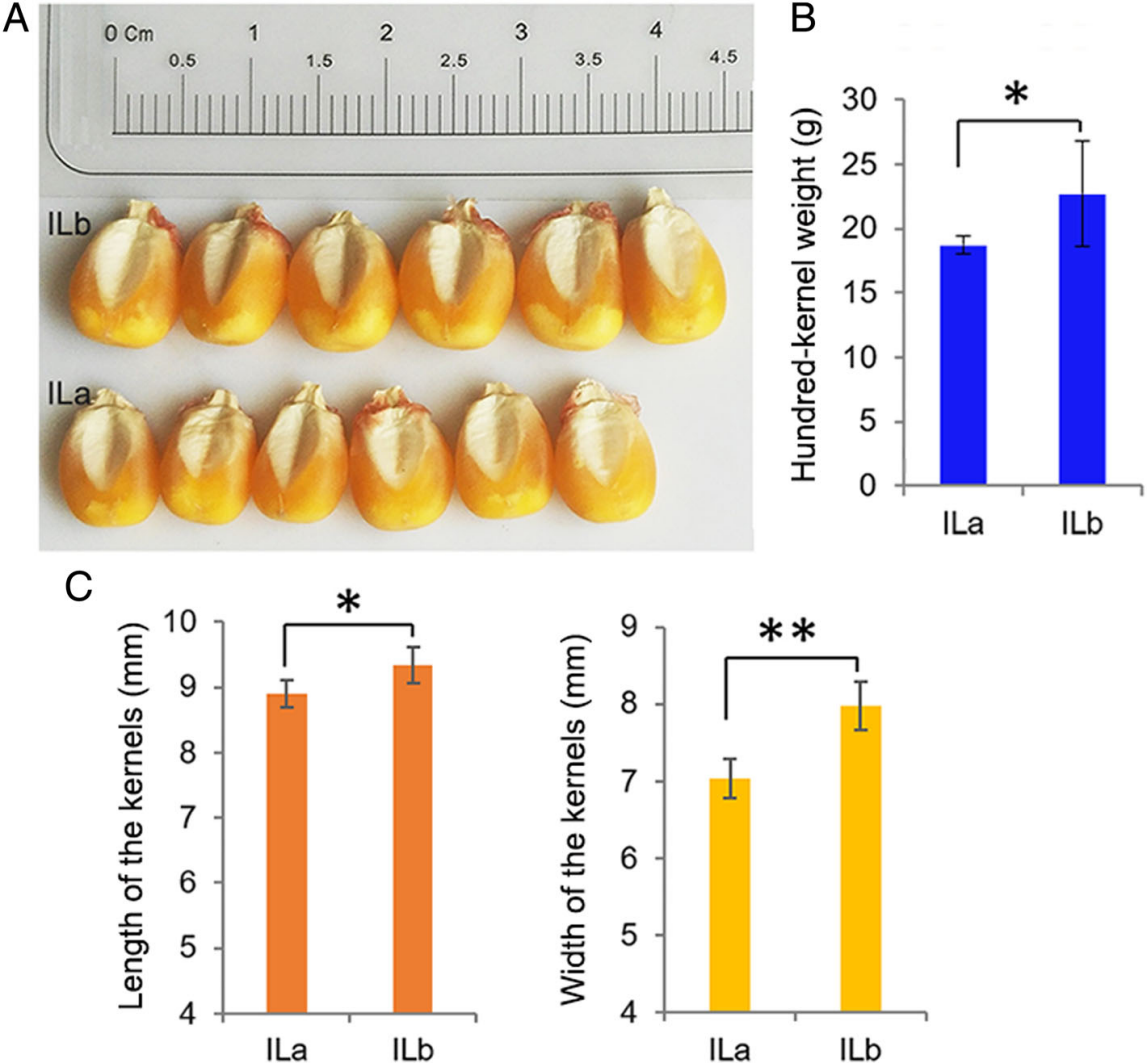
The final kernel size and hundred-grain weight (HGW) of ILa mature dehydrated kernel was significantly smaller than that of the ILb. (**a**) Phenotypes of mature dehydrated kernels of ILb and ILa. (**b**) The HGW of ILb is 21.56% more than that of ILa. (**c**) The different kernel length and width of the two derived inbreds. Error bars show the standard error value. The asterisk shows a significant difference between the two inbreds (*, *P* < 0.05; **, *P* < 0.01) by Student’s t-test

Two maize lines, ILa (JN14–7-22) and ILb (JN14–7-13), were developed from a single F_2_ progeny ear of a self-crossed hybrid after another three rounds of self-crossing. The mature dry kernel size of ILa selfing progenies was smaller than that of ILb kernels. The kernel length and width of ILa were smaller than those of ILb kernels. The one hundred-kernel-weight (HKW) of ILa kernels was significantly lower than that of ILb; the HKW of ILb was 21.56% more than that of ILa (Fig. 1). While the mature plant heights of ILa and ILb were similar (Additional file 2: Figure S1).

**Fig. 2:**
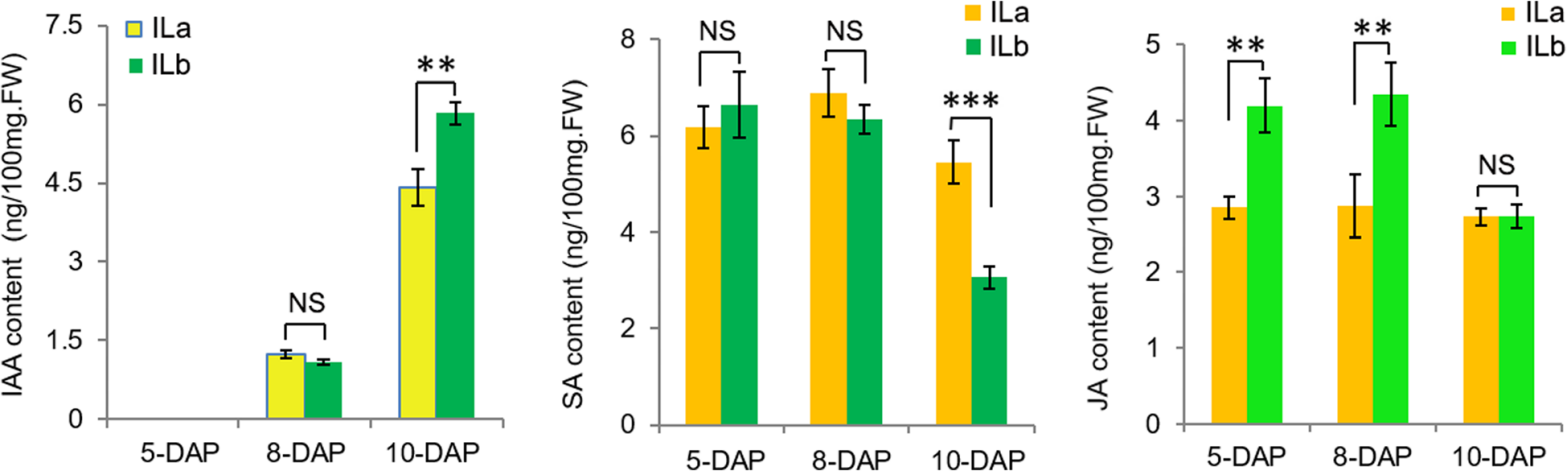
Phytohormone levels in the early young kernel of ILa and ILb by HPLC. In the 5-DAP kernels, no IAA could be detected and abundant SA and JA could be detected; in the 5-DAP and 8-DAP kernels, only JA displayed significant difference between ILa and ILb. However, in the 10-DAP kernels, both IAA and SA level displayed significant difference between ILa and ILb and the previous JA level difference between ILa and ILb diminished. Data represent average of three biological replicates. Error bars show the standard error value. Values are the mean ± SD. The asterisk shows a significant difference between the two inbreds (**, *P* < 0.01, ***, *P* < 0.001) by Student’s t-test; NS, not significant

To investigate the possible role of phytohormones in determining kernel size during early kernel development in maize, the levels of the most important growth and defense hormones (IAA, SA, and JA) were measured in ILa and ILb kernels at 5, 8, and 10 DAP. At 5 DAP, no IAA was detected in the kernels of either inbred. At 8 DAP, IAA was detected in low abundance in kernels of both inbreds; the IAA level was slightly higher in ILa kernels than in ILb kernels. At 10 DAP, the IAA level was significantly lower in ILa kernels than in ILb kernels. SA was detected at relatively similar high abundance in ILa and ILb kernels at 5 and 8 DAP; at 10 DAP, SA was significantly higher in ILa kernels than in ILb kernels due to a sharp reduction of SA in ILb kernels and a relatively small reduction of SA in ILa kernels. JA was significantly lower in ILa kernels than in ILb kernels at 5 and 8 DAP, it was stable in developing ILa kernels at all three timepoints (Fig. 2). These results indicate that the significantly lower IAA level and higher SA level at 10 DAP in ILa kernels may correlate with the smaller kernel size and HKW of ILa kernels, compared to that in ILb kernels.

### ILa and ILb kernel transcriptomes significantly differed at 5 and 8 DAP

We investigated the gene expression networks during early kernel development in ILa and ILb to evaluate potential molecular mechanisms regulating phytohormone levels and mature kernel sizes. Developing kernels were collected at 5, 8, and 10 DAP from field-grown ILa and ILb plants for comparative transcriptome profiling by performing principal component analysis (PCA). Striking differences were observed in ILa and ILb kernel transcriptomes at 5 and 8 DAP. The ILa kernel transcriptome at 5 DAP (a5) clustered closely to the ILb kernel transcriptome at 8 DAP (b8), suggesting that the temporal developmental progression was faster in ILa kernels than in ILb kernels. The ILa kernel transcriptome at 8 DAP (a8) and the ILb kernel transcriptome at 5 DAP (b5) were strikingly different from the other kernel transcriptomes. By contrast, the ILa kernel transcriptome at 10 DAP (a10) clustered closely to the ILb kernels at 10 DAP (b10), indicating that the difference between ILa and ILb kernels at 10 DAP was reduced (Additional file 3: Figure S2).

**Fig. 3:**
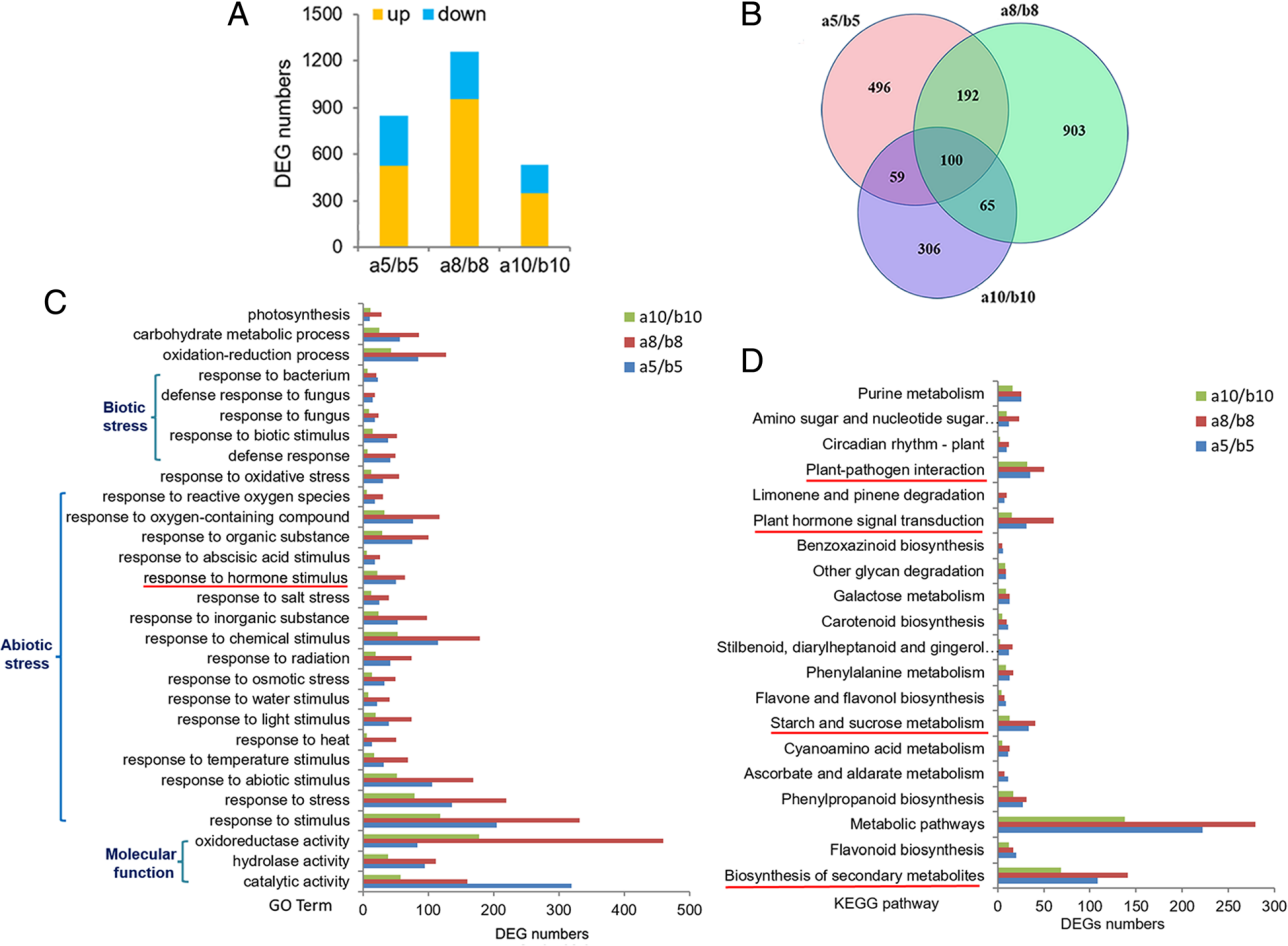
The global differences in the dynamics of the transcriptome in the early developing kernels between ILa and ILb. (**a**) Total DEG numbers from the three transcriptome pairs between ILa and ILb at different developing time. The total DEG number peaked in a8/b8 (1261) and down to the lowest in a10/b10 (530) transcriptome pair, more than half the DEGs were up-regulated in all the three (a5/b5, a8/b8 and a10/b10) transcriptome pair. a5 or b5 is the transcriptome from ILa or ILb kernel at 5 DAP, and so on for a8, b8 and a10, b10. (**b**) Small amount of DEGs overlapped between/among the different transcriptome pairs between ILa and ILb. (C) GO analysis indicates that most of the DEGs from the three transcriptome pairs were enriched in biological processes like defense response to biotic stress and abiotic stress, with molecular function like catalytic or hydrolase or oxidoreduction activity. Significant difference in “response to hormone stimulus” was observed between ILa and ILb at 5 DAP and 8 DAP. (D) KEGG pathway enrichment analysis with the DEGs from the three transcriptome pairs. Notably, ‘starch and sucrose metabolism’, ‘plant hormone signal transduction’ and ‘plant-pathogen interaction’ were all enriched with abundant DEGs from a5/b5, a8/b8 and a10/b10 transcriptome pairs

The parameters used for screening differentially expressed genes (DEGs) between ILa and ILb were fold change (FC) of the expression level in ILa [FC ≥2 or FC ≤0.5 under *P*-value ≤0.05, false discovery rate (FDR) ≤0.05)] compared to the expression level in ILb at the same developmental stage based on the sequenced fragments per kilobase of transcript per million mapped reads (FPKM). The transcriptional differences between ILa and ILb peaked at 8 DAP, as 1261 DEGs were found in the a8/b8 transcriptome pair, and 957 (76%) of these DEGs were upregulated in ILa kernels, compared to the expression levels in ILb kernels at 8 DAP. And 847 DEGs and 530 DEGs were obtained from the a5/b5 transcriptome pair and the a10/b10 transcriptome pair, respectively. Only 100 DEGs from these transcriptome pairs overlapped among all developmental stages; a5/b5 shared 292 and 159 DEGs with a8/b8 and a10/b10 transcriptome pairs, respectively. Only 165 DEGs overlapped between a8/b8 and a10/b10 transcriptome pairs (Fig. 3a, b). Of the overlapped 100 DEGs, the FPKM value of 47 DEGs were higher in ILa kernels at almost all time-points, including 16 DEGs whose FPKM value decreased to below 1 in ILb kernels after 8-DAP, and 35 DEGs were exclusively expressed in ILa kernels and 6 DEGs were exclusively expressed in ILb kernels at all time-points (Additional file 4: Figure S3). This indicates that almost all the overlapped 100 DEGs displayed similar direction of gene expression changes among different contrasts, except a few DEGs that showed opposite gene expression changes at 8- DAP or at 10-DAP. Gene ontology (GO) analysis indicated that most of these DEGs were enriched in biological processes related to responses to abiotic stress (temperature, heat, light, water, osmotic stress, chemical stimulus, salt, auxin, ABA, and ROS) or biotic stress (defense responses to fungi and bacteria). The enrichment of defense-related functional categories was consistent with the high levels of SA and JA in maize kernels at 5 and 8 DAP (Fig. 2). The top-enriched molecular functions were catalytic activity, hydrolase activity, and oxidoreductase activity (Fig. 3c). The a5/b5 and a8/b8 transcriptome pairs contained 54 and 68 DEGs, respectively, that encode components of ‘response to hormone stimulus’, indicating significant differences in hormone signaling in ILa and ILb kernels during this period.

The top-enriched KEGG (Kyoto Encyclopedia *of* Genes and Genomes) pathways of these DEGs were secondary metabolite biosynthesis pathways, including flavonoid, phenylpropanoid, flavone and flavonol, stilbenoid, diarylheptanoid and gingerol, and benzoxazinoid biosynthesis pathways. Other highly enriched pathways were metabolic pathways, including ascorbate and aldarate metabolism, starch and sucrose metabolism, and phenylalanine metabolism. Large number of DEG were enriched in ‘plant-pathogen interaction’ pathways at all timepoints. Abundant DEGs in a5/b5 and a8/b8 transcriptome pairs were enriched in ‘starch and sucrose metabolism’, ‘plant hormone signal transduction’; by contrast, these two pathways were remarkably reduced in DEG numbers in a10/b10 transcriptome pairs (Fig. 3d). Of the 61 DEGs that enriched in ‘plant hormone signal transduction’ from a8/b8, most of them expressed higher in ILa than that of in ILb at almost all the three time-points, including 7 of them specifically expressed in ILa kernels; except 17 of them expressed higher in ILb than that in ILa at almost all the time-points (Additional file 5: Figure S4). These results indicate that there are remarkable differences in hormone- and defense-related GO/KEGG functional categories in ILa and ILb kernels at 5 and 8 DAP, suggesting that phytohormone signaling and defense responses may function as molecular regulators mediating early maize kernel development.

### Differences in phytohormone signaling and defense response in ILa and ILb kernels peaked at 8 DAP

**Fig. 4:**
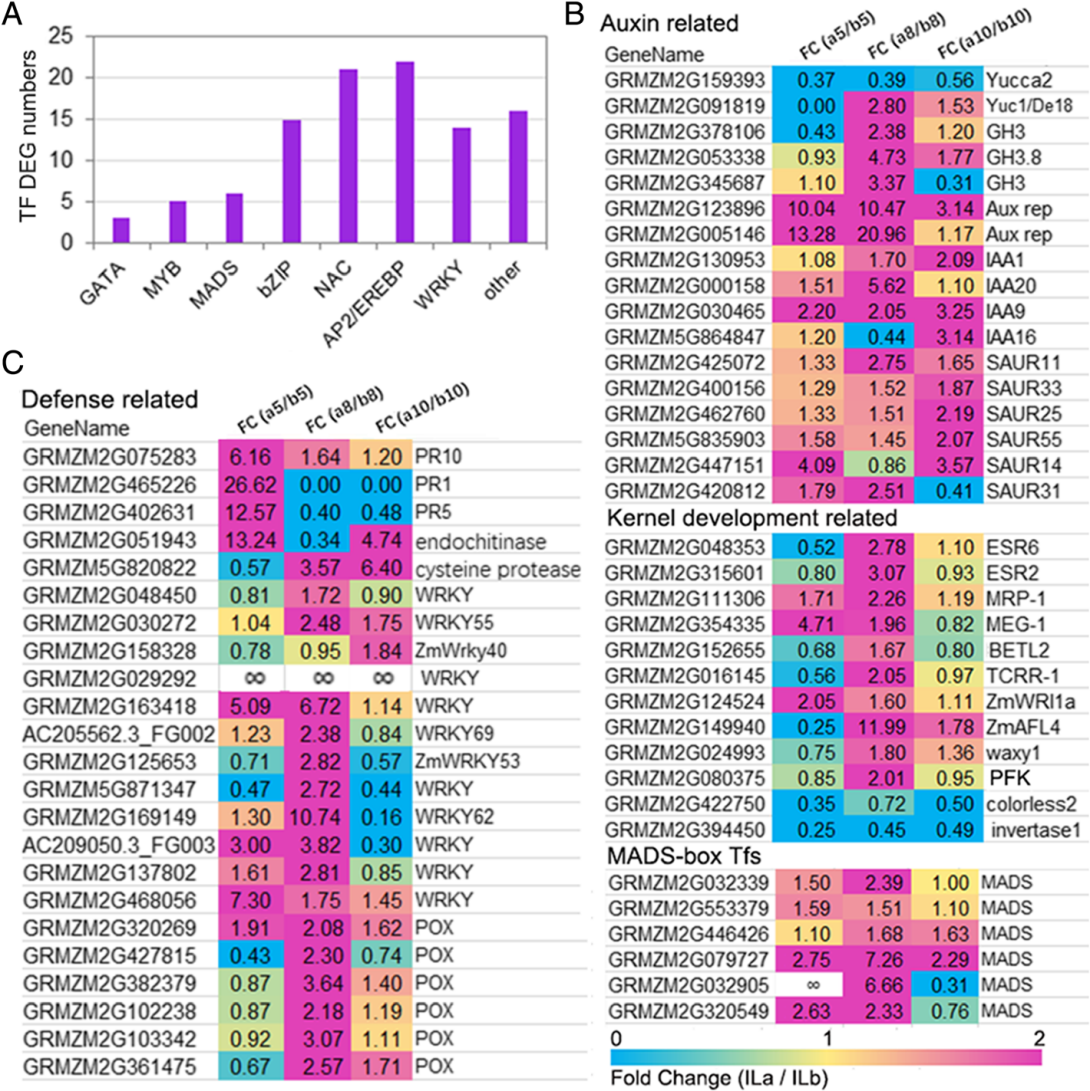
Expression profiles of the growth and defense related genes between ILa and ILb at different kernel developmental stages. (**a**) There were totally 102 transcription factors (TFs) that were differentially expressed (FC > 1.5 at FDR < 0.05) between ILa and ILb in 8-DAP developing kernels. These differentially expressed TFs were from 18 families, including the 5 MYB, 6 MADS, 15 bZIP, 21NAC, 22 AP2/EREBP, 14 WRKY, and 3 GATA family members. (**b**) The fold changes (FC) of the auxin-related, kernel development related, or the MADS TFs, based on the FPKM values in ILa and ILb at the three developmental stages. The expression level of most of these DEGs were remarkably higher in ILa transcriptomes than that in the respective ILb transcriptomes, except the DEGs that encoding Yucca2, colorless2 and invertase1, which were all down-regulated in all the ILa transcriptomes than that in the respective ILb transcriptomes. (**c**) The FC of the defense related DEGs, based on the FPKM in ILa and ILb at the three developmental stages. At 5 DAP, the expression levels of the genes encoding pathongensis-related proteins (PR) were specifically higher in ILa kernel than that in ILb kernel, but the difference reduced at 8 and 10 DAP; the expression level of WRKY member and the POX encoding genes were almost specially higher in ILa 8DAP kernel than that in ILb 8DAP kernel

Consistent with the above results, ILa and ILb kernels at 8 DAP had striking differences in the expression of certain transcription factor (TF) encoding genes, auxin-, kernel-development- and defense- related genes (Fig. 4). We identified 102 TF-encoding DEGs that displayed significantly higher expression levels in ILa transcriptomes than in the respective ILb transcriptomes, especially at 8 DAP. The TF-encoding DEGs were from 18 families, including MYB, MADS, bZIP, NAC, AP2/EREBP, WRKY, and GATA. The *Yuc1* expression level was significantly higher in a8 than in b8 kernels, whereas *Yucca2* expression was higher in b8 than in a8. The expression levels of genes encoding indole-3-acetic acid-amido synthetase (GH3), auxin-responsive Aux/IAA family members (IAA1, IAA20, IAA9), and auxin-responsive SAUR family members (SAUR11, SAUR33, SAUR25, SAUR55, SAUR31) were all significantly higher in a8 than b8 kernels. Two genes encoding auxin-repressed proteins (Aux rep) displayed significantly higher expression levels (> 10-fold) in a5 and a8 transcriptomes than in b5 and b8 transcriptomes. Of kernel development, the DEGs that encoded 6-phosphofructokinase (PFK), ESR6, ESR2, MRP-1, MEG-1, TCRR-1, ZmWRI1a, ZmAFL4, and wx1 had significantly higher expression levels in a8 than in b8 kernels. The transcript levels of *invertase1* and *colorless2* were significantly higher in ILb transcriptomes at all times than that in the respective ILa transcriptomes (Fig. 4, Additional file 6: Figure S5, Additional file 1: Table S1). These indicate that ILb kernels have higher sucrose metabolism as invertases hydrolyze sucrose into glucose and fructose.

We detected 53 genes that encode WRKY family members; 11 of these displayed significantly higher expression levels in a8 than in b8 kernels. One WRKY member (GRMZM2G029292) was specifically expressed in ILa kernels. *PR10*, *PR1*, and *PR5* displayed significantly higher expression levels in a5 than in b5 kernels; *PR5* expression level was > 10-fold higher in a5 kernels (276.72) than in b5 kernels (22.01) (Fig. 4). Other defense-related DEGs, including six *peroxidase* genes, four *GST* genes, and 18 *CYP* genes, all displayed significantly higher expression levels in a8 than in b8 kernels (Additional file 1: Table S1). These highly expressed DEGs involved in auxin, kernel development, or defense-response in a5 and a8 kernels may contribute to difference in early kernel development compared to b5 and b8 kernels and lead to smaller mature kernel size in ILa compared to ILb.

Some growth-related and defense-related genes showed dramatically abrupt changes in expression levels in ILa 8 DAP kernel. The expression levels of growth-related genes, encoding expansin, sucrose synthase1, et al., dramatically and abruptly decreased to almost zero in ILa 8 DAP kernels, and then recovered to similar levels as in ILb 10 DAP kernels. The expression levels of defense-related genes, encoding sulfur-rich/thionin-like protein, T22K18_16, hydrophobic protein RCI2B, senescence-associated protein DIN1, and natterin-4 dramatically, et al., abruptly increased in a8 kernels, and then decreased to similar levels as ILb kernels at 10 DAP (Additional file 7: Figure S6). These dramatically abrupt changes in expression levels suggest rapid global transcriptional reprogramming and an imbalance in growth and defense response in ILa kernels at 8 DAP, this might be associated with the simultaneous higher IAA and SA levels in ILa kernels at 8 DAP, compared to that of ILb kernels.

### Dramatic transcriptional reprogramming occurred earlier in ILa than in ILb during early kernel development

**Fig. 5:**
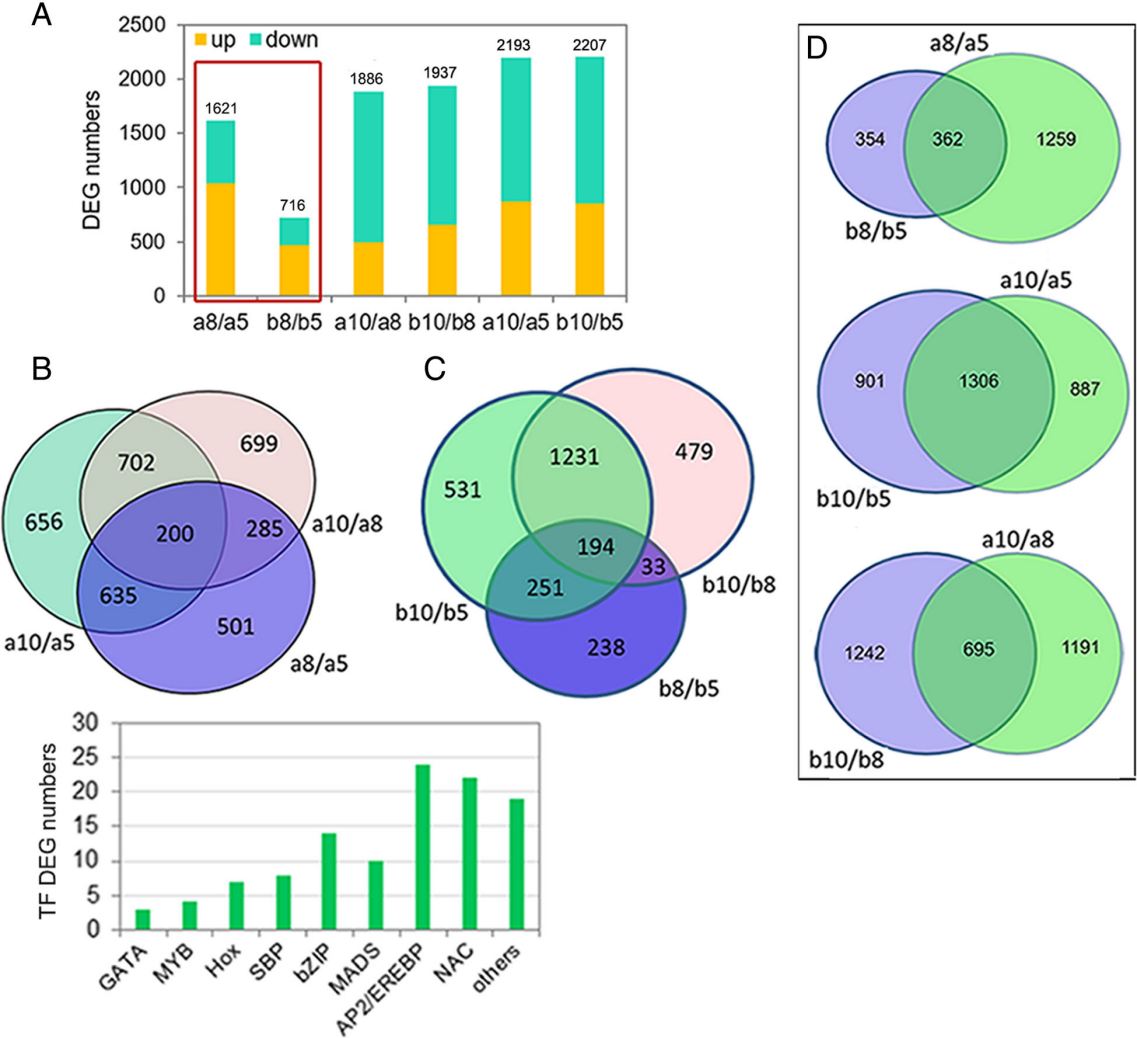
The transcriptome reprogramming is different in ILa kernel or ILb kernel during early development stage. (**a**) The obtained DEG numbers from different transcriptome pairs at different developing stage of ILa or ILb. a8/a5 or b8/b5 means transcriptome from ILa or ILb kernel collected at 8-DAP compared to transcriptome from ILa or ILb kernel collected at 5-DAP, and so on for a10/a5, b10/b5, a10/a8, b10/b8. (**b**-**d**) The overlapping of the DEGs between /among the transcriptome pairs from ILa (B) or ILb (**c**) at different developing stage or between ILa and ILb at the same stage (**d**). (**e**) 111 TF-encoding genes were detected to be differentially expressed at least in one of the three transcriptome pairs of ILa or ILbs. Multiple Hox and SBP, while no WRKY encoding genes were found to be differentially expressed in the three transcriptome pairs of ILa or ILb

ILa and ILb kernels displayed striking differences in their transcriptional profiles at 8 DAP, and they also showed their own specific transcription profiles from 5-DAP to 10-DAP. During kernel development from 5 to 8 DAP, the number of DEGs in ILa (1621 DEGs in the a8/a5 transcriptome pair) was more than twice the number of DEGs in ILb (716 DEGs in the b8/b5 transcriptome pair). The number of DEGs in the other two transcriptome pairs did not significantly differ; there were 1886 and 1937 DEGs in the a10/a8 and b10/b8 transcriptome pairs, respectively; and 2193 and 2207 DEGs in the a10/a5 and b10/b5 transcriptome pairs, respectively (Fig. 5a). These results indicate that dramatic transcriptional reprogramming in ILa kernels occurs during the transition from 5 to 8 DAP, whereas dramatic transcriptional reprogramming in ILb kernels occurs during the transition from 8 to 10 DAP. Within the ILa transcriptome pairs, a8/a5 shared more than half of its DEGs (835) with a10/a5, but only shared 485 of its DEGs with a10/a8, and a10/a5 shared 902 DEGs with a10/a8. The ILb transcriptome pairs had similar overlapping DEGs; b8/b5 shared 445 and 227 DEGs with b10/b5 and b10/b8, respectively, whereas b10/b5 shared 1425 DEGs with b10/8. During kernel development from 5 to 8 DAP, a8/a5 shared only 362 DEGs (22.33%) with b8/b5, with 1259 (77.67%) DEGs specifically owing to the a8/a5 transcriptome pair. From 8 to 10 DAP, transcriptome pair a10/a8 shared 695 (36.85%) DEGs with the b10/b8 transcriptome pair, each with almost two- thirds DEGs specific to its transcriptome pair, whereas a10/a5 shared more than half of its DEGs (1306) with b10/b5 from 5 to 10 DAP (Fig. 5b–d). A total of 111 TF-DEGs were differentially expressed in at least one of the three transcriptome pairs of ILa or ILb. We detected multiple DEGs encoding Hox and SBP TFs, whereas no *WRKY* members were differentially expressed in ILa or ILb at any developmental stage (Fig. 5e). These suggest that the greatest difference in transcriptional profiles between ILa and ILb is observed during the transition from 5 to 8 DAP. The dramatic transcriptional reprogramming induced a faster rate of developmental progression, which may begin at 5 DAP in ILa and 8 DAP in ILb, and this substantially different developmental processes ultimately contribute to morphological differences in ILa and ILb kernels.

### The onset of the significantly enriched functional categories was earlier in ILa young kernel at 5 DAP but weaker at 10 DAP, compared to that in ILb young kernel

**Fig. 6:**
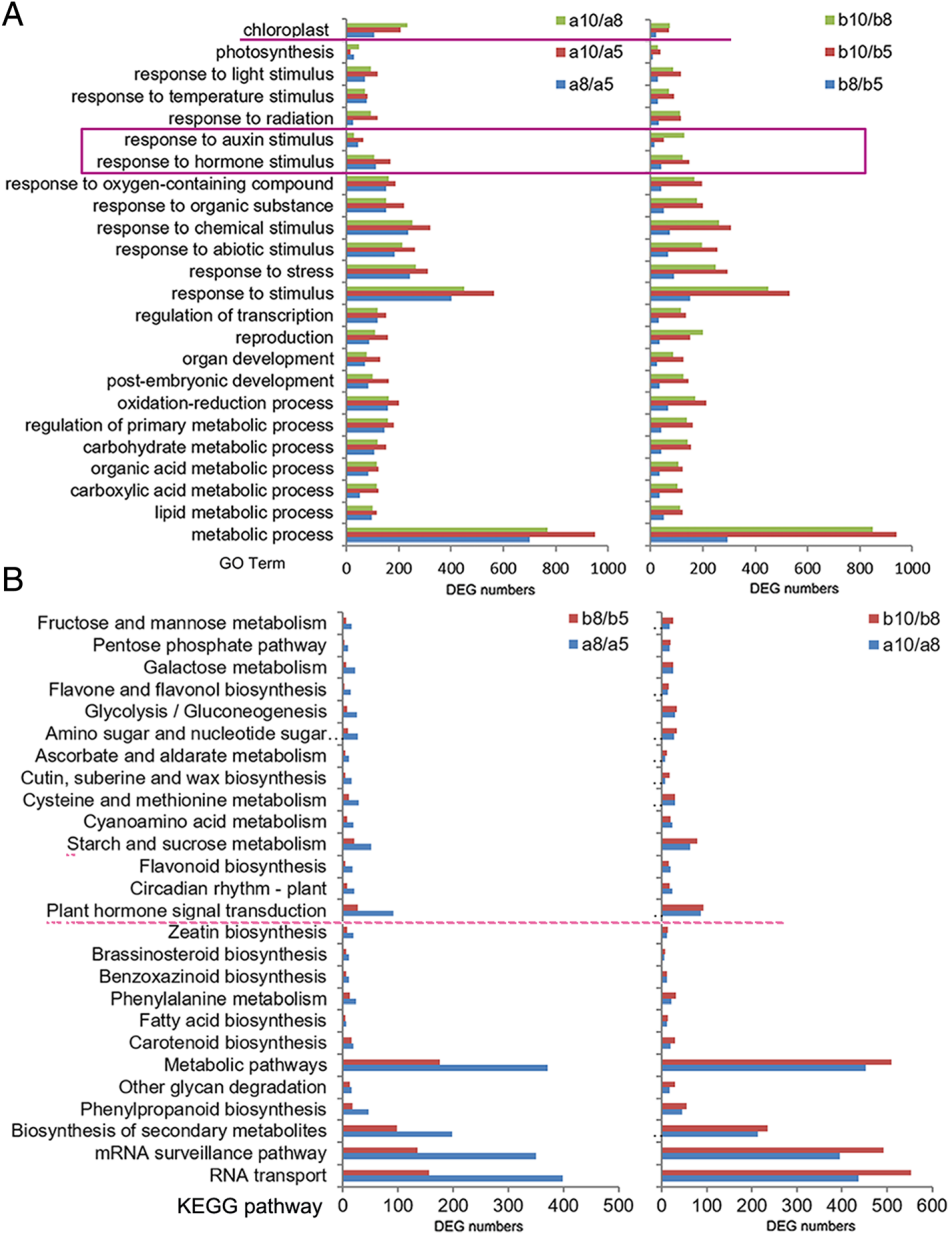
The earlier onset of the significantly enriched functional categories in ILa kernel than that in ILb kernel, by functional analysis of the DEGs from different transcriptome pair during early kernel development stage of ILa and ILb. (**a**) The DEGs from different transcriptome pair at different development stage showed similar GO term enrichment. Much more DEGs were enriched in all the top-enriched terms in a8/a5 than that in b8/b5 transcriptome pair, especially the term “response to auxin stimulus”, “response to hormone stimulus”. (**b**) The top-enriched KEGG pathways from different transcriptome pair at different kernel development stage of ILa and ILb. The biggest difference in transcriptome reprogramming between ILa and ILb is at 5 to 8 DAP, there are 3 pathways, “Starch and sucrose metabolism”, “plant hormone signal transduction” and “phenylpropanoid biosynthesis”, displayed significant enrichment in ILa but not in ILb at 5 to 8 DAP. While the DEGs that were enriched in all the top-enriched pathways from a10/a8 were less than that from b10/b8 transcriptome pair

Functional analysis of DEGs from transcriptome pairs of ILa or ILb at different developmental stages showed that the a8/a5 transcriptome pair contained significantly more DEGs in all the top-enriched GO categories (biotic and abiotic stress responses) than the b8/b5 transcriptome pair. By contrast, and the enriched DEG number difference between a10/a8 and b10/b8 transcriptome pairs was not significant in most of these GO categories. Striking differences in the numbers of DEGs from ILa and ILb were observed in the GO category “response to auxin stimulus”, in which a8/a5 contained 44 DEGS, b8/b5 contained 15 DEGs; while a10/a8 contained 29 DEGs, and b10/b8 contained 130 DEGs. Similar trends were observed for “response to hormone stimulus”, “reproduction” and “post-embryonic development”. ILa transcriptome pairs (a10/a8 and a8/a5) contained more DEGs enriched in “chloroplast” than the respective ILb transcriptome pairs (b10/b8 and b8/b5) (Fig. 6a). Consistently, the a8/a5 transcriptome pair contained more DEGs that were enriched in all the top-enriched KEGG pathways than the b8/b5 transcriptome pair. The b10/b8 transcriptome pair contained more DEGs that were enriched in all these top-enriched KEGG pathways than the a10/a8 transcriptome pair. DEGs that were enriched in the functional categories of “starch and sucrose metabolism”, “plant hormone signal transduction”, and “phenylpropanoid biosynthesis”, were all significantly more in a8/a5 than that in b8/b5 transcriptome pairs; conversely, DEGs that were enriched in these functional categories were significantly more in b10/b8 than in a10/a8 transcriptome pairs. For the “starch and sucrose metabolism” category, a8/a5 contained 52 DEGs, b8/b5 contained 20 DEGs; a10/a8 contained 63 DEGs, while b10/b8 contained 79 DEGs; this suggests that starch and sucrose metabolic pathways are more active during 5 to 8 DAP and less active at later stages from 8 to 10 DAP in ILa kernels, compared to that in ILb kernels. For the “plant hormone signal transduction” category, a8/a5 contained 91 DEGs, b8/b5 contained 27 DEGs; a10/a8 contained 87 DEGs, while b10/b8 contained 93 DEGs (Fig. 6b). These results indicate that the onset of significantly enriched functional categories was earlier in ILa (at 5 DAP), whereas these functional categories were similarly or more significantly enriched in ILb at later stages (8 DAP).

## Discussion

Maize is the world’s leading crop. The mature kernel size in maize is determined by the cooperative interactions of many maternal and zygotic factors during early kernel development. This suggests that molecular control of the termination of early kernel development is crucial for later developmental stages [33]. Many studies report that seed development is regulated by novel genes, transcriptional networks [34, 35]. Hormone signaling is a key regulatory mechanism that determines mature seed size [36, 37]. IAA accumulates in BETL, aleurone, and ESR just before maize endosperm starts to accumulate starch, high transcript and protein levels of the auxin transporter *ZmPIN1* are detected in BETL and ESR [38]. IAA levels undergo more than 50-fold accumulation in developing kernels during endosperm cellularization and early starch deposition [39].

### Phytohormones have important roles in maize early kernel development

During maize kernel development, the dramatic transcriptional reprogramming determines the rate of developmental progression, and phytohormone plays critical role in these important biological processes. We detected high levels of SA and JA, while no/low level of IAA in the maize young kernel before 8 DAP; IAA level remarkably increased and SA decreased in 10 DAP kernels. At 10 DAP, ILb kernels had significantly higher IAA levels than that of ILa kernels. IAA level is associated with total cell number and cell volume in developing maize kernel, as the large reductions in free IAA levels in mutant *de18* maize endosperm causes decreased total cell numbers and smaller cell volume in its endosperm [15]. The mitotic cell division phase during early kernel development is known largely responsible for generating the final population of endosperm cells [4]. The significantly higher IAA levels in ILb kernels at 10 DAP might result in the generation of more endosperm cells in ILb than in ILa kernels, which ultimately leads to larger mature kernel size in ILb.

SA and JA are associated with defense and stress responses, and they can antagonize other phytohormone pathways. IAA and SA biosynthetic and signaling pathways have antagonistic interactions. It is reported that *mn1* kernels have high SA levels and low IAA levels [40]. At 8 DAP, both IAA and SA levels in ILa kernels were slightly higher than that of ILb, while JA level in ILa kernels was significantly lower than that of ILb. This contradiction might be associated with the abrupt changes in expression levels of the growth-related and defense-related genes (imbalance in growth and defense response) in ILa 8 DAP kernels (Fig. 2, Additional file 7: Figure S6). At 10 DAP, significantly higher IAA and lower SA levels were detected in ILb kernels, compared to ILa kernels at 10 DAP. The abundant SA and JA levels in early maize kernels likely reflects complex crosstalk between hormone pathways and activation of general stress responses. Consistently, most of the top-enriched GO functional categories were defense response related, indicating these complex crosstalk plays important roles in early kernel development (Figs. 2, 3, 4, 5 and 6).

Complex crosstalk has been reported between sugar and hormone pathways in developing maize kernels. The carbohydrate status (hexoses-sucrose ratio) has a key role in maize seed size determination by controlling mitotic activity in the endosperm. Sugar levels could also possibly regulate IAA and SA biosynthesis independently and in opposite directions, reduced cell wall invertase activity in mutant *mn1* kernels has been associated with reduced IAA content and increased SA content compared to that in WT kernels [40]. So higher IAA and lower SA could be seen in ILb kernel at 10-DAP, compared to that in ILa-10-DAP kernel. The *invertase1* is a key gene for maize kernel development [40], its transcript level in ILb kernels was always > 2-fold greater than that in the respective ILa kernels at all timepoints (Fig. 4b). Prolonged higher expression of *invertase1* may result in a higher hexoses-sucrose ratio. This ratio acts as a metabolic signal for extended mitotic activity in ILb kernels, more cells lead to more starch granules, and subsequently this positively correlates with larger mature kernels and greater seed weight. Sugar levels also regulate IAA and SA biosynthesis independently and inversely, leading to higher IAA and lower SA in ILb kernels at 10 DAP compared to that in ILa kernels at 10 DAP (Fig. 2).

### The transcriptional differences between ILa and ILb kernels peaked at 8 DAP with abundant top-enriched defense related functional categories

PCA identified significant global differences in ILa and ILb transcriptome dynamics at 5 to 8 DAP; these transcriptional differences peaked at 8 DAP and reduced at 10 DAP (Fig. 3, Additional file 3: Figure S2). The ILa kernel transcriptome at 5 DAP clustered closely to the ILb kernel transcriptome at 8 DAP, while ILa and ILb transcriptomes were clustered closely at 10 DAP (Fig. 3a, Additional file 3: Figure S2). Essentially all of the top-enriched GO functional categories between ILa and ILb at 5 and 8 DAP were defense responses to biotic and abiotic stress (Fig. 3c), suggesting that defense related functional categories are the biggest difference between ILa and ILb kernels during this stage. The component of “response to hormone stimulus” is enriched in DEG from a5/b5 and a8/b8 transcriptome pairs, although there is no significant difference in IAA and SA levels between ILa and ILb at 5 and 8DAP, while JA levels is significantly different between them during this stage. Different expression levels of genes associated with auxin (GH3, IAA, SAUR), kernel development (ESR, MRP-1, BETL), defense (PR-, POX, Cyt, GST) and TF-encoding genes (MADS, WRKY), peaked at 8 DAP in ILa and ILb kernels (Fig. 4). TFs are crucial for many important processes, such as WRKY family protein in the universal signaling pathways involved in responses to external stimuli [41]. *WRKY* family members are differentially expressed between ILa and ILb transcriptome pairs, whereas no *WRKY* members were differentially expressed, and multiple development-related Hox−/SBP TFs were differentially expressed within ILa or ILb transcriptome pairs during early kernel development (Figs. 4c and 5e). Enrichment of these functional categories is consistent with the high levels of SA and JA in 5 and 8 DAP maize kernels.

### Functional categories related to phytohormone signaling, development, and carbohydrate metabolism are initiated earlier in ILa

Grain filling has earlier onset, slower rate, and earlier termination in maize inbred lines with small seed compared to inbred lines with large seed [33]. Developmental progression is thought to be a critical factor in these seed weight differences, as small seeds display a linear increase in seed weight between 10 and 12 DAP (with faster developmental progression) and large seeds display this linear increase between 16 and 18 DAP [33]. The DEG number from a8/a5 transcriptome pair was > 2 fold than that of b8/b5 pair (Fig. 5a), indicating more dramatic transcriptional reprogramming and a faster rate of developmental progression in ILa kernels during 5 to 8 DAP. Functional categories such as ‘reproduction’ and ‘post-embryo development’, which are indispensable for normal kernel development, were initiated earlier in ILa kernels during 5 to 8 DAP (a8/a5) and was weaker in ILa kernels during 8 to 10 DAP (a10/a8), compared to those observed in the respective ILb kernels (b8/b5 and b10/b8) (Fig. 6a). This is consistent with a report that the onset of key genes was earlier in small seeds, while similar maximum transcription levels were observed in large seeds at later stages [33]. These results suggest that dramatic transcriptional reprogramming and faster developmental progression may begin in ILa kernels at 5 DAP and in ILb kernels at 8 DAP. These differences in developmental processes may ultimately contribute to differences in kernel development and mature kernel size.

Among the significantly enriched KEGG functional categories, ‘starch and sucrose metabolism’, ‘plant hormone signal transduction’, and ‘phenylpropanoid biosynthesis’ were more significantly enriched in the a8/a5 than in b8/b5 transcriptome pair and later more significantly enriched in the b10/b8 than in a10/a8 transcriptome pair. This suggests that these categories have earlier onset (at 5 DAP) and weaker activity (at 10 DAP) in ILa kernels than in the respective ILb kernels. These three pathways were also significantly enriched in a5/b5 and a8/b8 transcriptome pairs, further confirming these differences in transcriptional reprogramming during kernel development between ILa and ILb (Figs. 3d and 6b). Maize endosperm storage product synthesis and accumulation begins at ∼10 DAP. The earlier enrichment of ‘starch and sucrose metabolism’ in a8/a5 and its weaker activity in a10/a8 transcriptome pairs suggest that developing ILa kernels have an earlier onset of carbohydrate metabolism, at the time when ILb kernels are undergoing the intense mitotic cell division phase, which ultimately generates the final population of endosperm cells [4]. Significantly higher IAA levels in ILb kernels at 10 DAP, different transcriptional profiles in ILa and ILb during 5 to 10 DAP, and prolonged high expression of *invertase1* in ILb kernels (Figs. 2, 3, 4), leading to extended mitotic activity and cell proliferation, all indicate that ILb kernels may have a longer period of cell division and generate more endosperm cells, more starch granules, and ultimately larger mature kernel size and seed weight than ILa kernels. And later onset (after 8 DAP) of the significantly enriched KEGG functional categories, especially the ‘starch and sucrose metabolism’, ‘plant hormone signal transduction’ categories, coincide with stronger activity of these categories at a later developmental stage (10 DAP), are necessary for the young kernel to undergo longer mitotic activity, produce more cells, and finally develop a larger kernel size. The different onset times and complex interactions of the important functional categories, especially phytohormone signal, and carbohydrate metabolism, form the most important molecular regulators mediating differences in maize early kernel development and mature kernel size.

## Methods

### Plant materials and samples preparation

Two maize derived inbreds, ILa (JN14–7-22) and ILb (JN14–7-13), were developed from a single F_2_ progeny ear of a self-crossed hybrid to reduce background genetic differences. Their progenies were further self-crossed for another three rounds and selected for further experiments of this study. At the beginning, these progenies that had similar appearances, such as their mature plant height, ear cob colors, kernel colors and, mature ear harvest time, et al., were selected for further analysis. Later, these progenies were selected when they had different final kernel size. The final kernel size differences were further confirmed for two other rounds of self-crossing generation and then the lines were selected and planted for the following experiments. Then, the selected lines were planted and self-crossed. The primary ears were collected from 5 to 10 self-crossed field-growing plants (2016 in Beijing) at indicated time-points, 5 DAP, 8 DAP and 10 DAP, and only the kernels at the medium part of the ear were collected at each developing time-point (stage) of each inbred, and used for different biological experiment repeats for phytohormone and transcriptome profile analysis. Each line we sampled 10 well-pollinated ears and only kernels from the medium part of the ear were selected for the hundred-kernel-weight (HKW) and had 30 kernels from those used for the HKW measurement for the kernel length and width measurement.

### Phytohormone quantification

All the young kernels used for IAA, SA and JA quantification were sampled from 3 different ears and used for different biological experiment repeats. The collected kernels were immediately frozen in liquid and stored at − 80 °C for further experiments. The IAA, JA, and SA contents were quantified using ultra-high-pressure liquid chromatography-tandem mass spectrometry (UHPLC/MS-MS). The standards were purchased from Sigma-Aldrich (St. Louis, MO, USA) and the internal standard for IAA was D_2_-IAA (Sigma-Aldrich). Sample preparation was performed using solid-phase extraction using a C-bound 18 silica column on the basis of reversed-phase interaction; liquid chromatography was carried out using a UFLC with an autosampler (Shimadzu Corporation, Kyoto, Japan). Experimental details were performed as described according to Liu et al. [42]. Differences in phytohormone concentration between ILa and ILb were tested using a two-way ANOVA, and followed by a *t*-test (*P* < 0.05). A *P* < 0.05 value indicated significant correlation between kernel phytohormone level and kernel genotype difference in the tested population.

### RNA-seq and detection of differentially expressed genes (DEGs)

To investigate the molecular mechanism that underlying the different phytohormone levels and different final kernel sizes of the two maize inbreds, young developing kernels were collected at 5-DAP, 8-DAP and 10-DAP from ILa and ILb plants in the field (2016 in Beijing) for mRNA-sequencing. The kernels used for mRNA-sequencing were collected from the same ears that were sampled for phytohormone detection or mRNA-sequencing. The collected young kernels were immediately frozen in liquid nitrogen and stored at − 80 °C until RNA extraction. Total RNA extraction and Poly(A) RNA isolation were performed according to the manufacturer’s protocol (Invitrogen). RNA-seq libraries were prepared according to the Illumina standard instructions (TruSeq Standard RNA LT Guide), followed by a quality check with the Agilent 2100 bioanalyzer, and sequenced on the Illumina HiSeq 3000 platform according to the manufacturer’s instructions (HiSeq 3000 User Guide) by Nanjing Vazyme-Bio, in order to generate 150 bp paired-end reads. Effective reads were aligned to Ensembl plants release-30 zea_−_mays genome build Zea_mays.AGPv3.26 (http://plants.ensembl.org/Zea_mays/Info/Index). The unique reads were normalized as reads per kilobase of exon model per million mapped reads using Samtools v0.1.19 [43]. Differentially expressed genes (DEGs) were defined as those with the fold change (FC) of the expression level (FC ≥2 or FC ≤0.5 under *P*-value ≤0.05, FDR ≤0.05) in certain transcriptome compared to the expression level in the control transcriptome. GO enrichment and KEGG enrichment (KEGG enrichment/pathwat graph) were performed using the obtained DEGs. DEGs were defined Q value < 0.001.

### Statistical analysis

Statistical analysis was performed with the paired Student’s *t*-test. All values represent the mean ± SD. *P < 0.05; **P < 0.01; ***P < 0.001.

## Additional files

Additional file 1: **Table S1**. Fold changes of the expression of auxin-, kernel development- and defense- related genes and certain transcription factor (TF) encoding genes during ILa and ILb early kernel development. (XLSX 42 kb)

Additional file 2: **Figure S1**. The field-grown mature plant height of ILa and ILb inbred was similar. This data was obtained from two years (in Beijing at 2015 and 2016) (TIF 152 kb)

Additional file 3: **Figure S2**. The global differences in the transcriptome dynamics during early kernel development between ILa and ILb at different developmental stage by principal component analysis (PCA) analysis. The ILa 5 DAP kernel transcriptome (a-5DAP) clustered closely to ILb 8 DAP transcriptome (b-8DAP), the a-8DAP and b-5DAP kernels were strikingly different from the other kernels, while ILa and ILb was clustered very closely at 10 DAP. a-5DAP, a-8DAP, a-10DAP are ILa kernel transcriptome at 5 DAP, 8 DAP, 10 DAP, respectively; b-5DAP, b-8DAP, b-10DAP are ILb kernel transcriptomes at 5 DAP, 8 DAP, 10DAP, respectively. (TIF 600 kb)

Additional file 4: **Figure S3**. Heatmap of the overlapped 100 DEGs from different contrasts. Almost all the DEGs displayed similar direction of gene expression changes that were shared among different contrasts. (A) The FPKM value of 47 DEGs were dramatically higher in ILa kernels than that in ILb kernels at all time-points, and the FPKM value of 16 DEGs (the pink line indicated) decreased to below 1 in ILb kernels after 8-DAP. (B) 35 DEGs were exclusively expressed in ILa kernels at all time-points. (C) 11 DEGs (upper part) expressed higher in ILb kernels at almost all time-points and 6 DEGs (the pink line indicated) were exclusively expressed in ILb kernels at all time-points. The number is the FPKM value and to reduce the influence of transcription noise, here we defined a gene as expressed if its FPKM value was ≥1. (TIF 5941 kb)

Additional file 5: **Figure S4**. Heatmap of the 61 DEGs that enriched in ‘plant hormone signal transduction’ from a8/b8 transcriptome pair. (A) Most of them expressed higher in ILa than that of ILb at almost all the time-points, 7 of them specifically expressed in ILa kernels (pink line indicated). (B) 17 of them expressed higher in ILb than that of ILa at almost all the time-points. (TIF 1778 kb)

Additional file 6: **Figure S5**. Phytohormone levels and gene expression profiles in early development of the two kernels. (A) Profiles of the phytohormone levels in the two kernels. (B) Expression profiles of a few genes that is important in auxin biosynthetic and catabolic pathways in early development of the two kernels. (C) Expression profiles of a few genes that is important for maize kernel development. (TIF 2200 kb)

Additional file 7: **Figure S6**. The growth-related genes and the defense-related genes showed conversely abrupt expression changes in ILa 8 DAP kernels. Left, the growth-related genes, encoding expansin, sucrose synthase1, et al., were abruptly decreased to almost zero in ILa 8 DAP kernels and relatively recovered to similar to that of ILb at 10 DAP; right, the defense-related genes, encoding sulfur-rich/thionin-like protein, T22K18_16, hydrophobic protein RCI2B, senescence-associated protein DIN1, natterin-4, et al., were dramatically and abruptly elevated in ILa 8 DAP kernels and decreased to similar to that of ILb at 10 DAP. (TIF 4857 kb)

## Abbreviations

ABA: Abscisic acid
AL: Aleurone layer
BETL: Basal endosperm transfer layer
CKs: Cytokinins
CYPs: Cytochrome P450 proteins
DAP: Days after pollination
ESR: Embryo-surrounding region
GSTs: Glutathione-S-transferases
IAA: Indole-3-acetic acid
JA: Jasmonic acid
PR: Pathogenesis-related protein
ROS: Reactive oxygen species
SA: Salicylic acid
SE: Starchy endosperm

## References

[1] Dumas C, Rogowsky P (2008). Fertilization and early seed formation. C R Biol.

[2] Li G, Wang D, Yang R, Logan K, Chen H, Zhang S, Skaggs MI, Lloyd A, Burnett WJ, Laurie JD (2014). Temporal patterns of gene expression in developing maize endosperm identified through transcriptome sequencing. Proc Natl Acad Sci U S A.

[3] Leroux BM, Goodyke AJ, Schumacher KI, Abbott CP, Clore AM, Yadegari R, Larkins BA, Dannenhoffer JM (2014). Maize early endosperm growth and development: from fertilization through cell type differentiation. Am J Bot.

[4] Doll NM, Depège-Fargeix N, Rogowsky PM, Widiez T (2017). Signaling in early maize kernel development. Mol Plant.

[5] Rijavec T, Jain M, Dermastia M, Chourey PS (2011). Spatial and temporal profiles of cytokinin biosynthesis and accumulation in developing caryopses of maize. Ann Bot.

[6] Rijavec T, Kovac M, Kladnik A, Chourey PS, Dermastia M (2009). A comparative study on the role of cytokinins in caryopsis development in the maize miniature1 seed mutant and its wild type. J Integr Plant Biol.

[7] Lituiev DS, Krohn NG (2013). M€uller B, Jackson D, Hellriegel B, Dresselhaus T, Grossniklaus U. theoretical and experimental evidence indicates that there is no detectable auxin gradient in the angiosperm female gametophyte. Development..

[8] Chen J, Lausser A, Dresselhaus T (2014). Hormonal responses during early embryogenesis in maize. Biochem Soc Trans.

[9] Lur HS, Setter TL (1993). Role of auxin in maize endosperm development (timing of nuclear DNA endoreduplication, zein expression, and cytokinin). Plant Physiol.

[10] Phillips Kimberly A., Skirpan Andrea L., Liu Xing, Christensen Ashley, Slewinski Thomas L., Hudson Christopher, Barazesh Solmaz, Cohen Jerry D., Malcomber Simon, McSteen Paula (2011). vanishing tassel2Encodes a Grass-Specific Tryptophan Aminotransferase Required for Vegetative and Reproductive Development in Maize. The Plant Cell.

[11] LeClere S, Schmelz EA, Chourey PS (2008). Cell wall invertase-deficient miniature1 kernels have altered phytohormone levels. Phytochemistry..

[12] Zhao Y (2012). Auxin biosynthesis: a simple two-step pathway converts tryptophan to indole-3-acetic acid in plants. Mol Plant.

[13] Kim J, Baek D, Park H, Chun H, Oh D, Lee M, Cha J, Kim WY, Kim MC, Chung WS, Bohnert HJ, Lee SY, Bressan RA, Lee SW, Yun DJ (2013). Overexpression of Arabidopsis YUCCA6 in potato results in high-auxin developmental phenotypes and enhanced resistance to water deficit. Mol Plant.

[14] Li W, Zhao X, Zhang X (2015). Genome-wide analysis and expression patterns of the YUCCA genes in maize. J Genet Genomics.

[15] Bernardi J, Lanubile A, Li QB, Kumar D, Kladnik A, Cook SD, Ross JJ, Marocco A, Chourey PS (2012). Impaired auxin biosynthesis in the *defective endosperm18* mutant is due to mutational loss of expression in the ZmYuc1 gene encoding endosperm-specific YUCCA1 protein in maize. Plant Physiol.

[16] Naseem M, Kaltdorf M, Dandekar T (2015). The nexus between growth and defence signalling: auxin and cytokinin modulate plant immune response pathways. J Exp Bot.

[17] Goossens J, Fernández-Calvo P, Schweizer F, Goossens A (2016). Jasmonates: signal transduction components and their roles in environmental stress responses. Plant Mol Biol.

[18] Wasternack C, Hause B (2013). Jasmonates: biosynthesis, perception, signal transduction and action in plant stress response, growth and development. Ann Bot.

[19] Huang Huang, Liu Bei, Liu Liangyu, Song Susheng (2017). Jasmonate action in plant growth and development. Journal of Experimental Botany.

[20] Kidd BN, Kadoo NY, Dombrecht B, Tekeoglu M, Gardiner DM, Thatcher LF, Aitken EAB, Schenk PM, Manners JM, Kazan K (2011). Auxin signaling and transport promote susceptibility to the root-infecting fungal pathogen *Fusarium oxysporum* in *Arabidopsis*. Mol Plant-Microbe Interact.

[21] Clarke JD, Volko SM, Ledford H, Ausubel FM, Dong X (2000). Roles of salicylic acid, jasmonic acid, and ethylene in *cpr*-induced resistance in *Arabidopsis*. Plant Cell.

[22] Zhang Y, Goritschnig S, Dong X, Li X (2003). A gain-of-function mutation in a plant disease resistance gene leads to constitutive activation of downstream signal transduction pathways in *suppressor of npr1-1, constitutive 1*. Plant Cell.

[23] Huot B, Yao J, Montgomery BL, He SY (2014). Growth–defense tradeoffs in plants: a balancing act to optimize fitness. Mol Plant.

[24] Kazan K, Lyons R (2014). Intervention of phytohormone pathways by pathogen effectors. Plant Cell.

[25] Qi L, Yan J, Li Y, Jiang H, Sun J, Chen Q (2012). *Arabidopsis thaliana* plants differentially modulate auxin biosynthesis and transport during defense responses to the necrotrophic pathogen *Alternaria brassicicola*. New Phytol.

[26] Baxter A, Mittler R, Suzuki N (2014). ROS as key players in plant stress signalling. J Exp Bot.

[27] Wirthmueller L, Maqbool A, Banfield MJ (2013). On the front line: structural insights into plant-pathogen interactions. Nat Rev Microbiol.

[28] Luo Meng, Brown Robert L., Chen Zhi-Yuan, Menkir Abebe, Yu Jiujiang, Bhatnagar Deepak (2011). Transcriptional Profiles Uncover Aspergillus flavus-Induced Resistance in Maize Kernels. Toxins.

[29] Dickman MB, Fluhr R (2013). Centrality of host cell death in plant-microbe interactions. Annu Rev Phytopathol.

[30] Alessandra Lanubile, Luca Pasini, Adriano Marocco (2010). Differential gene expression in kernels and silks of maize lines with contrasting levels of ear rot resistance after Fusarium verticillioides infection. Journal of Plant Physiology.

[31] Qu J, Ma C, Feng J, Xu S, Wang L, Li F, Li Y, Zhang R, Zhang X, Xue J, Guo D (2016). Transcriptome dynamics during maize endosperm development. PLoS One.

[32] Feng Fan, Qi Weiwei, Lv Yuanda, Yan Shumei, Xu Liming, Yang Wenyao, Yuan Yue, Chen Yihan, Zhao Han, Song Rentao (2018). OPAQUE11 Is a Central Hub of the Regulatory Network for Maize Endosperm Development and Nutrient Metabolism. The Plant Cell.

[33] Sekhon RS, Hirsch CN, Childs KL, Breitzman MW, Kell P, Duvick S, Spalding EP, Buell CR, de Leon N, Kaeppler SM (2014). Phenotypic and transcriptional analysis of divergently selected maize populations reveals the role of developmental timing in seed size determination. Plant Physiol.

[34] Kohl S, Hollmann J, Blattner FR, Radchuk V, Andersch F, Steuernagel B, Schmutzer T, Scholz U, Krupinska K, Weber H (2012). A putative role for amino acid permeases in sink-source communication of barley tissues uncovered by RNA-seq. BMC Plant Biol.

[35] Thiel J (2014). Development of endosperm transfer cells in barley. Front Plant Sci.

[36] Schruff MC, Spielman M, Tiwari S, Adams S, Fenby N, Scott RJ (2006). The AUXIN RESPONSE FACTOR 2 gene of *Arabidopsis* links auxin signalling, cell division, and the size of seeds and other organs. Development..

[37] Jiang WB, Huang HY, Hu YW, Zhu SW, Wang ZY, Lin WH (2013). Brassinosteroid regulates seed size and shape in Arabidopsis. Plant Physiol.

[38] Forestan C, Meda S, Varotto S (2010). ZmPIN1-mediated auxin transport is related to cellular differentiation during maize embryogenesis and endosperm development. Plant Physiol.

[39] Abu-Zaitoon YM, Bennett K, Normanly J, Nonhebel HM (2012). A large increase in IAA during development of rice grains correlates with the expression of tryptophan aminotransferase OsTAR1 and a grain-specific yucca. Physiol Plant.

[40] LeClere S, Schmelz EA, Chourey PS (2010). Sugar levels regulate tryptophan-dependent auxin biosynthesis in developing maize kernels. Plant Physiol.

[41] Jiang Y, Liang G, Yang S, Yu D (2014). Arabidopsis WRKY57 functions as a node of convergence for jasmonic acid- and auxin-mediated signaling in jasmonic acid-induced leaf senescence. Plant Cell.

[42] Liu H, Li X, Xiao J, Wang S (2012). Convenient method for simultaneous quantification of multiple phytohormones and metabolites: application in study of rice-bacterium interaction. Plant Methods.

[43] Li H, Handsaker B, Wysoker A, Fennell T, Ruan J, Homer N, Marth G, Abecasis G, Durbin R (2009). 1000 genome project data processing subgroup. The sequence alignment/map format and SAMtools. Bioinformatics..

