# Supplementary figures and images for: Characterization of phytohormone and transcriptome reprogramming profiles during maize early kernel development
Source: BMC Plant Biol. 2019 May 14;19:197. doi: 10.1186/s12870-019-1808-9 (PMC6515667; doi:10.1186/s12870-019-1808-9)

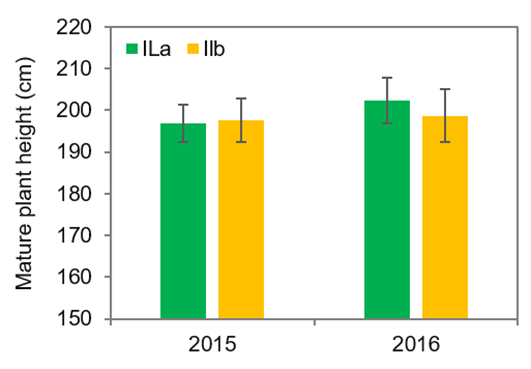

Supplement: Supplementary file 2 — Figure S1. The field-grown mature plant height of ILa and ILb inbred was similar. This data was obtained from two years (in Beijing at 2015 and 2016) (TIF 152 kb) [file 12870_2019_1808_MOESM2_ESM.tif]

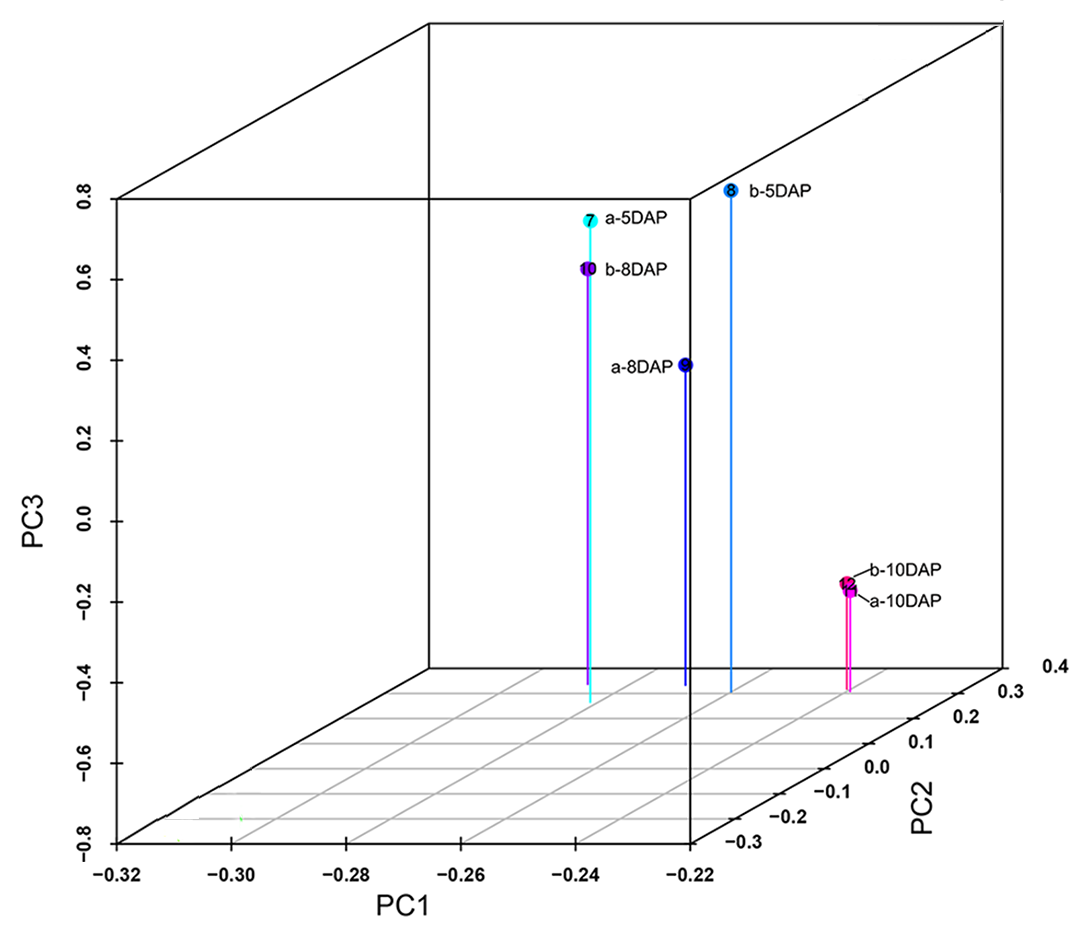

Supplement: Supplementary file 3 — Figure S2. The global differences in the transcriptome dynamics during early kernel development between ILa and ILb at different developmental stage by principal component analysis (PCA) analysis. The ILa 5 DAP kernel transcriptome (a-5DAP) clustered closely to ILb 8 DAP transcriptome (b-8DAP), the a-8DAP and b-5DAP kernels were strikingly different from the other kernels, while ILa and ILb was clustered very closely at 10 DAP. a-5DAP, a-8DAP, a-10DAP are ILa kernel transcriptome at 5 DAP, 8 DAP, 10 DAP, respectively; b-5DAP, b-8DAP, b-10DAP are ILb kernel transcriptomes at 5 DAP, 8 DAP, 10DAP, respectively. (TIF 600 kb) [file 12870_2019_1808_MOESM3_ESM.tif]

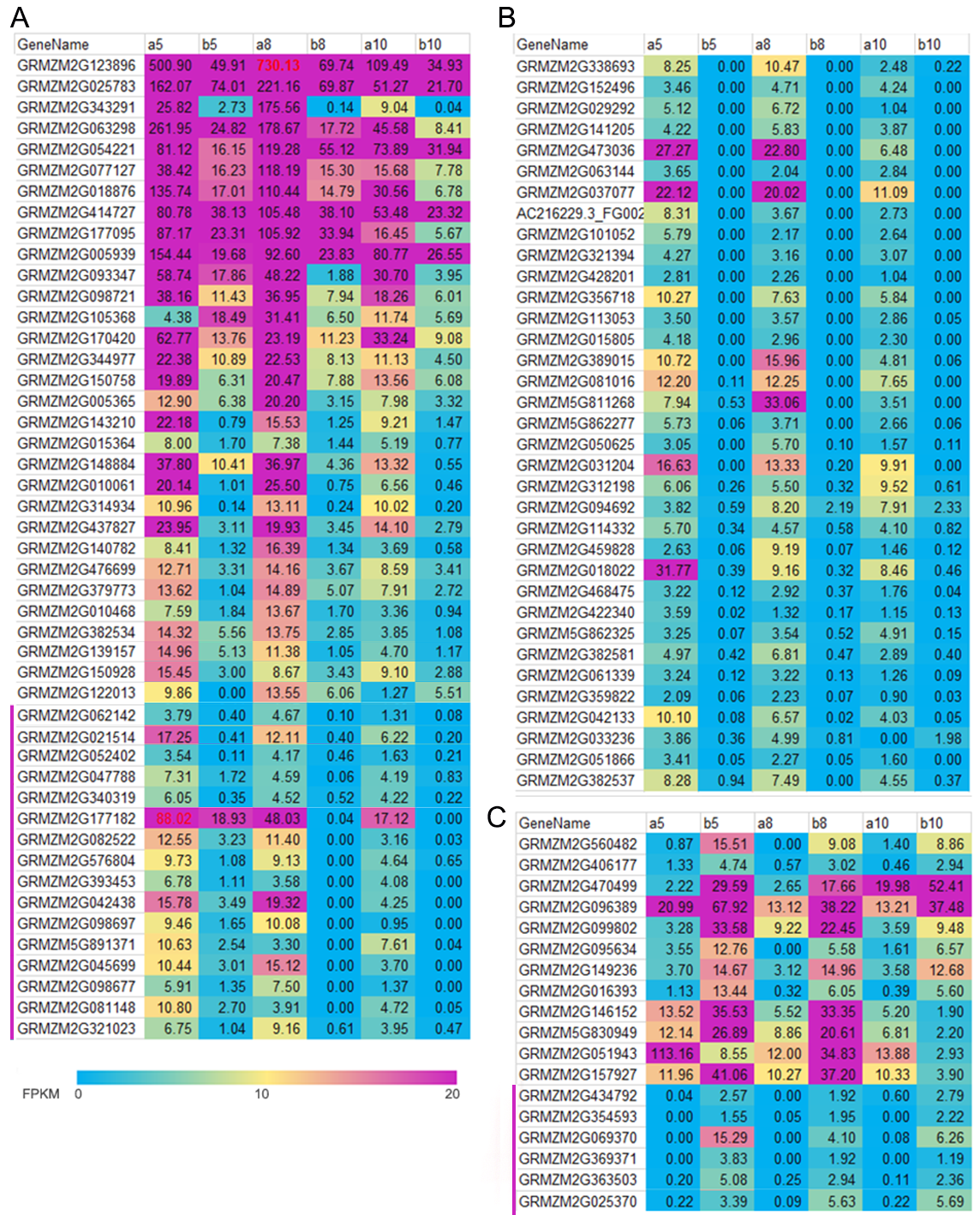

Supplement: Supplementary file 4 — Figure S3. Heatmap of the overlapped 100 DEGs from different contrasts. Almost all the DEGs displayed similar direction of gene expression changes that were shared among different contrasts. (A) The FPKM value of 47 DEGs were dramatically higher in ILa kernels than that in ILb kernels at all time-points, and the FPKM value of 16 DEGs (the pink line indicated) decreased to below 1 in ILb kernels after 8-DAP. (B) 35 DEGs were exclusively expressed in ILa kernels at all time-points. (C) 11 DEGs (upper part) expressed higher in ILb kernels at almost all time-points and 6 DEGs (the pink line indicated) were exclusively expressed in ILb kernels at all time-points. The number is the FPKM value and to reduce the influence of transcription noise, here we defined a gene as expressed if its FPKM value was ≥1. (TIF 5941 kb) [file 12870_2019_1808_MOESM4_ESM.tif]

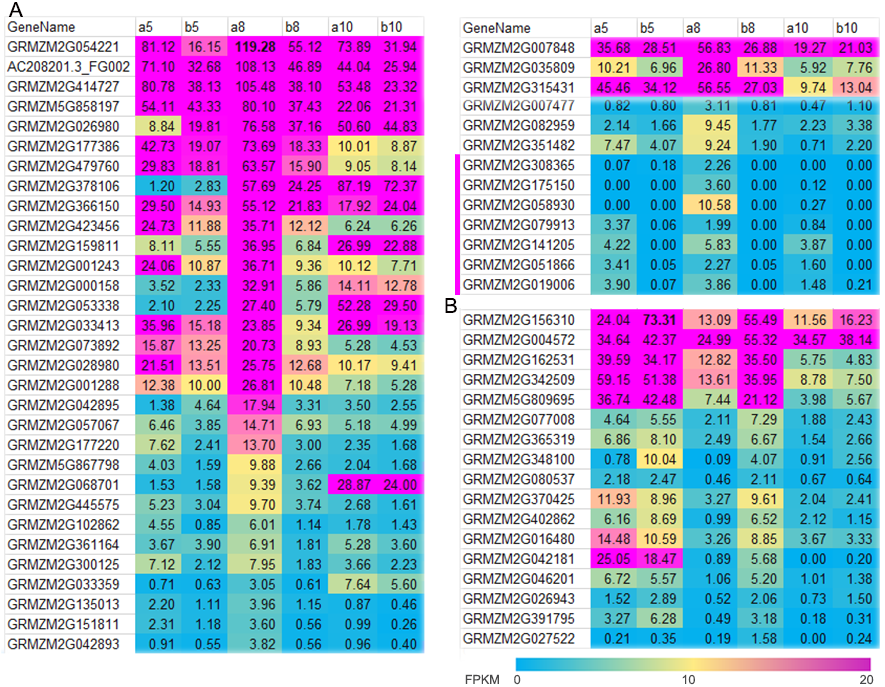

Supplement: Supplementary file 5 — Figure S4. Heatmap of the 61 DEGs that enriched in ‘plant hormone signal transduction’ from a8/b8 transcriptome pair. (A) Most of them expressed higher in ILa than that of ILb at almost all the time-points, 7 of them specifically expressed in ILa kernels (pink line indicated). (B) 17 of them expressed higher in ILb than that of ILa at almost all the time-points. (TIF 1778 kb) [file 12870_2019_1808_MOESM5_ESM.tif]

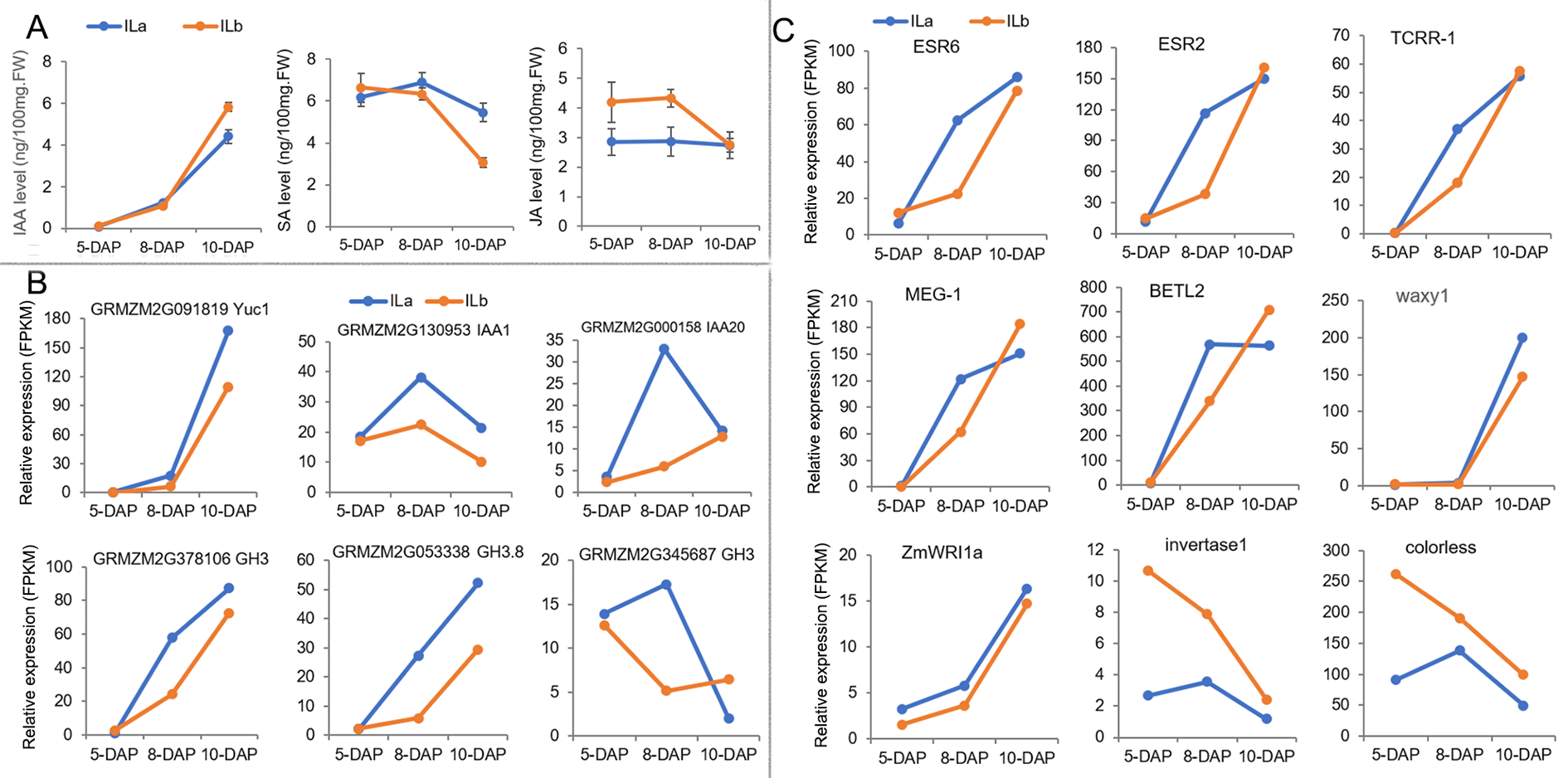

Supplement: Supplementary file 6 — Figure S5. Phytohormone levels and gene expression profiles in early development of the two kernels. (A) Profiles of the phytohormone levels in the two kernels. (B) Expression profiles of a few genes that is important in auxin biosynthetic and catabolic pathways in early development of the two kernels. (C) Expression profiles of a few genes that is important for maize kernel development. (TIF 2200 kb) [file 12870_2019_1808_MOESM6_ESM.tif]

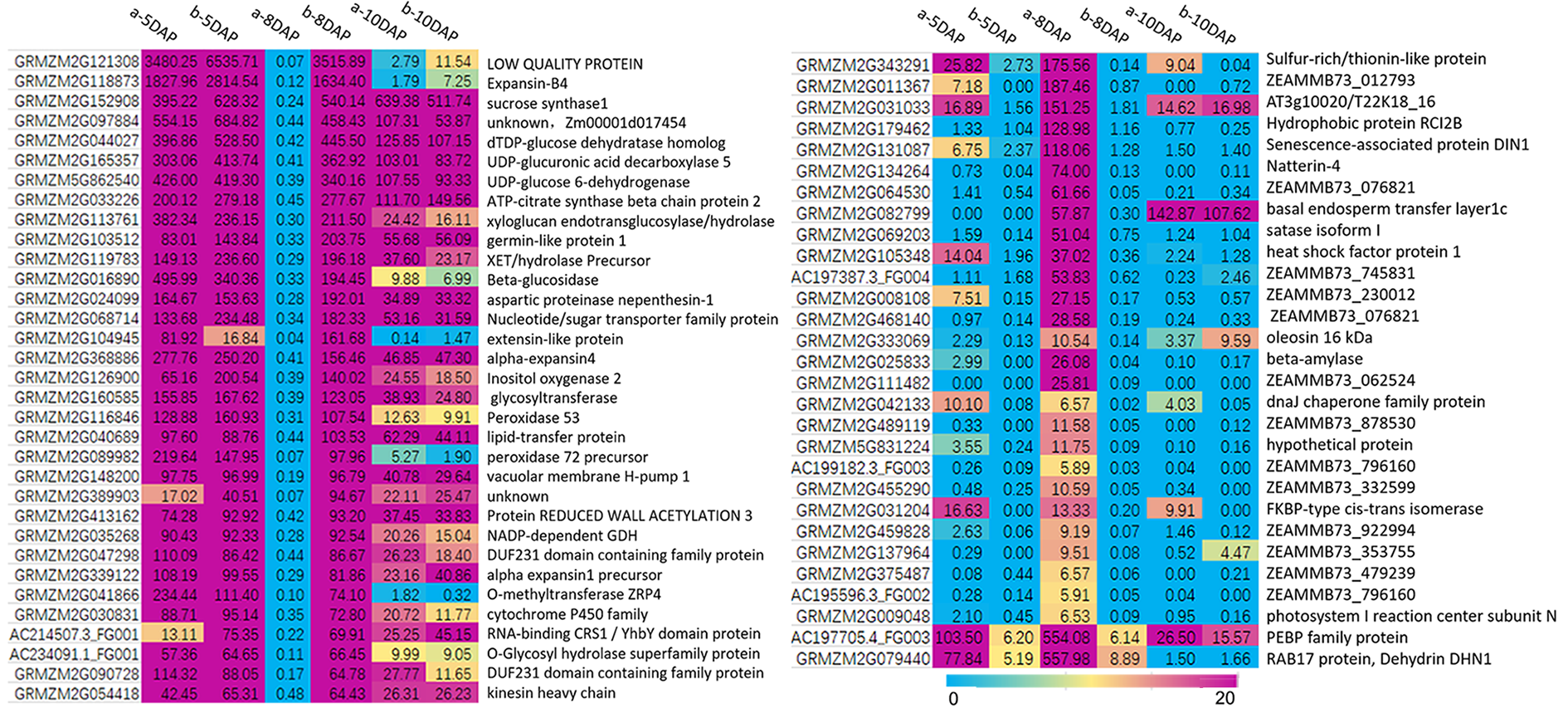

Supplement: Supplementary file 7 — Figure S6. The growth-related genes and the defense-related genes showed conversely abrupt expression changes in ILa 8 DAP kernels. Left, the growth-related genes, encoding expansin, sucrose synthase1, et al., were abruptly decreased to almost zero in ILa 8 DAP kernels and relatively recovered to similar to that of ILb at 10 DAP; right, the defense-related genes, encoding sulfur-rich/thionin-like protein, T22K18_16, hydrophobic protein RCI2B, senescence-associated protein DIN1, natterin-4, et al., were dramatically and abruptly elevated in ILa 8 DAP kernels and decreased to similar to that of ILb at 10 DAP. (TIF 4857 kb) [file 12870_2019_1808_MOESM7_ESM.tif]
